# Sequencing approaches in hereditary cancer testing: strengths, limitations and future directions

**DOI:** 10.1038/s41431-026-02078-x

**Published:** 2026-03-18

**Authors:** Sami Belhadj, Christopher J. Hatch, Holly LaDuca, Carolyn Horton, Seth I. Berger, Rachid Karam

**Affiliations:** Ambry Genetics, Aliso Viejo, CA USA

**Keywords:** Translational research, Cancer genetics, Genetics research, Medical genomics

## Abstract

Over the past three decades, Hereditary Cancer Testing (HCT) has evolved from single gene assays into multigene panel testing (MGPT), which allows for the screening of all known hereditary cancer genes in a single assay. MGPT is currently the standard approach for clinical HCT. However, with decreasing sequencing costs and increased instrument throughput, the scalability of exome sequencing (ES) and genome sequencing (GS) for HCT indications is becoming more viable. These methods provide broader insights into the coding exons and/or the entire genome, respectively. ES/GS data can also be reanalyzed to identify variants in novel genes that were not characterized at the time of initial testing, or to support research efforts aimed at uncovering additional associations between germline variants and cancer predisposition. Additionally, the emerging use of long-read sequencing (LRS) is noteworthy, enabling improved variant detection compared to short-read sequencing, especially for complex/structural variants and variation in difficult-to-sequence or paralogous regions in genes such as *PMS2*. This has the potential to increase the accuracy of HCT, reduce the turnaround time, find previously unidentifiable cancer risk variants, and ultimately increase the diagnostic yield. This article provides a comprehensive summary of the sequencing approaches used in HCT, discussing their strengths and limitations. We also highlight the added value of complementing DNA-only testing with RNA and tumor sequencing. Furthermore, we explore LRS-based approaches and discuss opportunities for their implementation in routine genetic testing for hereditary cancer.

## Introduction

The field of clinical cancer genetics has been evolving ever since the discovery and characterization of the first cancer predisposition genes (CPG) in the 80s–90s [1]. In the past, Hereditary Cancer Testing (HCT) consisted of single gene assays based primarily on Sanger sequencing to interrogate the protein coding regions and intron/exon boundaries of CPG such as *APC*, *BRCA1/2* and the mismatch repair (MMR) genes *MLH1* and *MSH2* [2, 3].

With the emergence of Next Generation Sequencing (NGS, i.e., second generation sequencing) during the late 2000s, sequencing cost and scalability improved drastically, allowing the development of assays covering multiple CPG simultaneously. In parallel, researchers have continued uncovering novel gene-disease associations raising the number of bona fide CPG to >100 genes [4]. Starting from the mid-2010s, multigene panel testing (MGPT) became the standard HCT approach and is widely used by molecular diagnostic laboratories around the world, who continue to expand and refine their gene menu following novel gene discoveries and/or new evidence supporting or refuting previously established associations [5]. Moreover, some clinical laboratories are complementing DNA-only MGPT with RNA testing to provide additional insights into the splicing effects of germline variation, and its association with increased cancer risk [6]. Similarly, tumor-normal sequencing allows the robust identification of somatic alterations to guide treatment decisions and may contribute additional information in the evaluation of germline variants, particularly variants of uncertain significance (VUS) [7, 8].

The falling cost of sequencing has also impacted Exome Sequencing (ES) and Genome Sequencing (GS), with modern ultra-high-throughput instruments capable of generating >1000 exomes or >100 genomes per run, at a considerably lower cost compared to a decade ago. For HCT, this could potentially translate into ES/GS replacing MGPT, as this creates an opportunity for data reanalysis over time, and powers research studies evaluating non-coding variation, polygenic risk scores (PRS) and novel CPG discovery [9, 10].

Alongside the evolution of NGS, Long Read Sequencing (LRS, i.e., third generation sequencing) exhibited remarkable improvements over the past five years, resulting in lower error rates, cost, and higher instrument throughput. Compared to short-read sequencing (SRS), LRS detects over 50% more structural variants (SV) and can directly identify epigenetic marks like 5-methylcytosine that influence gene expression [11]. It also enables phasing of variants in recessive cancer syndromes. These capabilities position LRS as a valuable tool in HCT—initially as an orthogonal method for complex regions like *PMS2*, and potentially as a future first-tier sequencing approach.

## DNA multigene panel testing

DNA MGPT were introduced commercially for hereditary cancer in 2012; however, widespread adoption did not begin until 2013 following the U.S. Supreme Court’s ruling to reverse the patent held on *BRCA1/2*. This decision enabled laboratories to offer comprehensive HCT via a single assay. In a study of individuals with breast and ovarian cancer, the proportion of HCT orders attributable to MGPT increased from ~40% at the end of 2013 to >90% by 2019 [12]. The average number of genes tested has also increased over time, with a trend toward larger pan-cancer panels over smaller, phenotype-specific panels [12, 13].

The clinical advantages of a DNA MGPT approach have been well established. Several large MGPT studies have demonstrated a significant increase in diagnostic yield over single gene/syndrome testing across cancer types. The diagnostic yield for MGPT is highly dependent upon the number of genes tested, indication for testing, and cohort selection. Initial results from commercial laboratories offering small-to-mid size panels (i.e., 25–34 genes) reported an overall diagnostic yield of 7–9% [13–15]. Subsequent studies of unselected cancer cohorts tested with larger panels (i.e., >75 genes) reported an overall diagnostic yield in the range of 13–17%, with the highest diagnostic yield observed among individuals with ovarian and pancreatic cancers [16, 17]. Other benefits of DNA MGPT include the identification of individuals with pathogenic/likely pathogenic variants (PV) in multiple CPG, in genes outside the initial testing indication, and refinement of penetrance estimates as a result of genotype-first characterization [18]. Based on these clinical advantages, expert guidelines recommend a MGPT approach to CPG testing [19].

Commercial laboratories generally perform MGPT using short-read targeted sequencing panels. The target enrichment of CPG is achieved by biotinylated probes that are designed to bind and capture regions of interest within CPG, primarily covering coding exons and intron-exon boundaries. Laboratories can also supplement their probe designs to capture regions of well-known deep intronic PV, including small variants and/or SV breakpoints [20]. Copy-number variants (CNV), mainly deletions and duplications, are inferred primarily from variations in expected read depth but may require orthogonal validation methods such as Multiplex Ligation-dependent Probe Amplification (MLPA) or microarrays. With the exception of exon-level deletions, CNVs can be reliably detected if read depth is sufficient. Of note, CNV calling algorithms are also evolving and display variable performance, with tools like GATK-gCNV showing high performance in MGPT data across CNV of different lengths [21].

The operational advantage of MGPT is cost effectiveness and scalability, as it allows laboratories to run hundreds-thousands of samples per day at a reduced cost compared to ES or GS (Table 1). MGPT also offers a higher sequencing depth for the desired targets, typically around 500x, which facilitates germline variant identification, as well as detection of mosaicism or clonal hematopoiesis of indeterminate potential [22].

**Table 1:** Summary of sequencing approaches in hereditary cancer testing (HCT).

Sequencing Approach	Targeted Sequencing / MGPT		ES	GS	
Genome coverage	10s–100s genes		All coding genes	Genome-wide	
Sequencing Technology	SRS	LRS	SRS	SRS	LRS
Current application level	Clinical (Standard)	Emerging	Clinical	Clinical	Emerging
Relative Cost	+	++	++	+++	++++
Sequencing depth^a^	~200–1000x	~50–200x	~100x	~30–40x	~20–40x
Covered regions^b^	Exons + splice regions	Exons + Introns + UTR	Exons + splice regions	Genome-wide^f^	Whole genome
Primary research potential	Candidate CPG	Candidate CPG, non-coding variants, SV, Epigenetics	Candidate CPG	Candidate CPG, non-coding variants, PRS	Candidate CPG, non-coding variants, PRS, SV, Epigenetics
**Variant Detection**					
SNV/indels ( < 50 bp)	✓	✓	✓	✓	✓
SV/CNV	Limited	✓	Limited	Limited	✓
Challenging HCT genes (*PMS2*)	Limited	✓	Limited	Limited	✓
Orthogonal confirmation required^c^	Yes	No	Yes	Yes	No
Epigenetics^d^	X	PCR: X; Native DNA: ✓	X	X	✓
**+ RNA Sequencing**					
Detection of abnormal splicing	✓	✓	✓	✓	✓
Detection of large intronic retentions	Limited	✓	Limited	Limited	✓
Isoform quantification	X	✓	X	X	✓
RNA quality requirements^e^	Medium	High	Medium-High	Medium-High	High
**+ Somatic Testing**					
Somatic second hits	✓	✓	✓	✓	✓
Mutational signature inference	X	X	✓	✓	✓
Epigenetics^d^	X	PCR: X; Native DNA: ✓	X	X	✓

*CPG* cancer predisposition genes, *ES* exome sequencing, *GS* genome sequencing, *HCT* hereditary cancer testing, Indel: small insertions and deletions ( < 50 bp), *LRS* Long-read sequencing, *MGPT* multigene panel testing, *PRS* polygenic risk scores, *SNV* single nucleotide variants, *SRS* short-read sequencing, *SV* Structural variants, *UTR* Untranslated regions.

^a^Depends on size of targeted regions and multiplexing.

^b^Denotes typically included regions.

^c^Orthogonal confirmation methods may include: Sanger sequencing, Multiplex Ligation-dependent Probe Amplification (MLPA), Long-range PCR sequencing or other techniques used by laboratories to validate suspected variants/alterations.

^d^Excluding the detection of low-level signals, such as mosaic methylation, where the limited sequencing depth translates into lower detection sensitivity.

^e^RIN (RNA Integrity Number) requirements vary by assay, typically RIN ≥ 5 for SRS, and RIN ≥ 7 for LRS applications.

^f^Coverage excludes known challenging SRS regions, such as repetitive and segmentally duplicated regions.

While MGPT has offered significant advantages over single-gene testing with respect to efficiency and identification of germline cancer predisposition, there are opportunities for improvement. One of the major limitations of targeted SR panels is the fixed design and subsequent need to regularly update the panel content. Professional testing guidelines are constantly evolving as novel CPG are discovered and established gene-disease relationships are refined. This, in turn, prompts laboratories to update their panels [19, 20]. While removing content could easily be achieved by bioinformatic masking, adding new content requires altering the probe design and necessitates clinical validation of the assay in each iteration, to ensure optimal performance in accordance with strict regulations of laboratory developed tests, such as the CLIA requirements in the US, and the IVDR in Europe.

There are also certain types of variants that are challenging to detect with targeted SR panels (Table 1). There are limitations in the ability to detect SV, especially when the breakpoints of these events are located outside the captured regions. For instance, copy-neutral events such as inversions and balanced translocations could go undetected if the breakpoints are not covered. Similarly, the inherent fluctuations of coverage uniformity in targeted sequencing might complicate the detection of smaller CNVs [23]. Another limitation of these assays is the accurate detection of variation in genes complicated by pseudogenes due to the shortcomings of SRS, prompting laboratories to validate suspicious findings with MLPA and long-range PCR [24].

## RNA sequencing as a complement TO HCT

RNA testing was traditionally performed with RT-PCR using primers overlapping exons followed by Sanger sequencing. The emergence of a more scalable approach - targeted RNA sequencing (RNA-seq) with hybridization capture – allows for the evaluation of multiple CPG in parallel. The utility of RNA-seq in HCT resides in providing qualitative and quantitative evidence to identify and characterize abnormal splicing events and aberrant expression levels [25]. Unlike other putative loss-of-function variants, most splicing variants are classified as VUS without additional functional evidence [26].

As a complement to HCT, RNA-seq provides added clinical value by facilitating the interpretation of splicing VUS and the detection of deep intronic splicing PV in regions that are not typically included in MGPT. This ultimately results in improved diagnostic resolution, along with a higher molecular diagnostic rate [6]. The first diagnostic study in a large consecutive series included >43,000 individuals undergoing paired DNA and RNA testing of 1–18 genes. Horton et al. reported a variant classification impact based on RNA evidence in 1.3% individuals (*n* = 547), including medically significant upgrades (0.2%, *n* = 70), novel deep intronic PV (0.1%, *n* = 27) and VUS downgrades (0.7%, *n* = 305) [27]. As many VUS are recurrent in the tested populations, these updated classifications were subsequently applied to a larger series of >500,000 previously tested individuals, yielding an impact on 4.5% of individuals (*n* = 22,963) with DNA-only testing, and where reclassifications were made in 33.1% of individuals (*n* = 7602) resulting in revised clinical reports sent to ordering clinicians. In another large laboratory-based cohort study involving paired DNA/RNA testing of 63 genes in >20,000 patients, 6.3% of individuals (*n* = 1279) had a non-VUS variant that would have been classified as a VUS without RNA evidence, including 0.4% with PV (*n* = 75), and 5.9% with benign or likely benign variants (*n* = 1204). Additionally, 0.2% (*n* = 40) of individuals had splicing PV that otherwise would not have been detected, as the variants were located outside the reportable range for the targeted panel [28].

RNA analysis also highlights the importance of assessing naturally occurring alternative splicing for clinical evaluation of variants in disease-causing genes [29]. For example, an analysis performed by the ENIGMA (Evidence-based Network for the Interpretation of Germline Mutant Alleles) Consortium indicated that *BRCA1* c.[594-2 A > C;641 A > G] (NM_007294), previously described to cause exon 10 skipping (a truncating alteration), displays characteristics inconsistent with those of a high risk pathogenic *BRCA1* variant [30]. Their RNA analysis of human samples and splicing minigenes showed that exon 10 skipping results from c.641 A > G altering splicing regulation, not from c.594-2 A > C disrupting the acceptor site. RNA assays revealed that while most transcripts were truncating, 20–30% were in-frame (Δ9,10) and predicted to encode a functional BRCA1 protein, indicating it should not be considered a high-risk pathogenic variant.

Another way to leverage RNA-Seq data is by performing allele specific expression analyses. Allelic expression levels can be altered by different mechanisms such as gene deletions, epigenetic silencing, cis-regulatory elements or loss-of-function variants that trigger non-sense mediated decay. These alterations could result in monoallelic expression, resulting from the complete silencing of one allele, or in allelic imbalances, which clinical significance is more challenging to determine depending on dosage sensitivity and the abundance of rescue transcripts that maintain sufficient function [31, 32].

The Clinical Genome Resource (ClinGen) Variant Classification Working Group has developed updated guidelines to clarify how the American College of Medical Genetics and Genomics (ACMG)/Association for Molecular Pathology (AMP) codes should be applied to splicing evidence [26]. The continued refinement and integration of these recommendations aim to standardize the interpretation of RNA and in-silico data, improving consistency in variant classification and expanding the clinical utility of RNA sequencing.

## Short read exome and genome sequencing

The cost to sequence a short read genome has dramatically declined over time, with sequencing costs expected to drop to $100–200/genome, consequently leading to a lower cost for ES. Similar to MGPT, the enrichment of all coding regions in ES is achieved by hybridization capture, whereas GS libraries are typically PCR-free and require less library preparation time. Required DNA amounts for ES and GS have also drastically decreased over time. These assays now require as low as 100–250 ng of gDNA, making them compatible with challenging specimen types that yield very limited amounts of gDNA, such as dried blood spots. Notably, there are now multi-omic GS assays that capture methylation data concurrently with genetic variation, leading to the detection of 5-methylcytosine and 5-hydroxymethylcytosine, such as Biomodal’s 6-base sequencing (Cambridge, UK), and Illumina’s 5-base sequencing (San Diego, CA, USA). These assays simplify the laboratory procedure to read the genome and epigenome without requiring multiple library preparation workflows, and without compromising the identification of C-to-T genetic variants [33].

As shown in Table 1, GS offers additional advantages over ES. The expanded coverage beyond exonic regions enables the identification of deep intronic variants and allows the delineation of SV breakpoints. For instance, there are two reported *PALB2* (NM_024675) Ex13 duplications, 4.8 kb and 13.8 kb, where only the smaller leads to abnormal RNA splicing and is of clinical relevance [34, 35]. MGPT or ES requires additional orthogonal testing to disambiguate the breakpoints in the absence of RNA testing. Additionally, GS allows the identification of non-coding alterations that could have an effect on splicing or gene regulation. While these variants are challenging to classify, the emergence of powerful new AI tools such as SpliceAI, PromoterAI and AlphaGenome in conjunction with RNA testing is expected to facilitate their interpretation [36–38]. Moreover, GS offers more uniform coverage compared to ES, especially in GC-rich regions, which facilitates CNV identification [39]. GS data can also be leveraged for PRS analysis, especially in patients of non-European ancestry. Notably, existing PRS were based on genome-wide association studies primarily from the European population, and consequently not suitable for clinical use in different populations [9]. In this context, the adoption of GS will guide PRS recalculation in underrepresented populations leading to more equitable and accurate cancer risk predictions.

The extent of genomic regions analyzed by MGPT, ES, and GS leads to substantial differences in average sequencing depth, typically around 500x for MGPT, 100x for ES, and 30x for GS, though these values can vary between laboratories. This variability directly impacts analytical performance, especially for read-depth-based CNV detection, where identifying small events that span only one or a few exons remains challenging [23]. Using 50x GS data, De La Vega et al. demonstrated that genome-wide CNV detection performance varies considerably depending on the CNV caller used, with sensitivity for smaller events (1–5 kb) generally lower, and duplications being detected less reliably than deletions [40]. Notably, performance improved when analyses focused on a virtual panel covering exons from 184 clinically relevant genes, including 89 CPG, after applying custom CNV filters and removing common artifacts. Ultimately, further research is needed to enable a direct comparison of the analytical performance of MGPT versus ES and GS for HCT, which will help clarify the benefits and trade-offs associated with each approach.

Interestingly, some laboratories are now creating hybrid GS-ES assays, to combine the higher sequencing depth of ES with the genome wide coverage of GS in a cost-effective manner. Boltz TA et al. devised and benchmarked the performance of a blended exome (30–40x mean depth) and low-pass genome (1-4x mean depth) sequencing assay against matched conventional ES, GS, or array data. The authors reported high recall and positive predictive value when calling coding CNV affecting ≥3 exons and high concordance for common variant imputation [41].

One of the main clinical advantages of ES and GS over MGPT is the opportunity for data reanalysis over time. This minimizes the need for sequential testing, as a patient’s sequencing data can be reanalyzed as phenotype evolves and as novel CPG and gene-disease relationships are discovered. ES and GS are the recommended first-tier testing approaches for patients diagnosed with rare disease. In contrast, the use of ES/GS in HCT has, for the most part, been limited to the research setting. Several CPG have been identified in studies leveraging ES/GS, such as *POLE, POLD1* and *NTHL1* [42, 43]. More recently, *MBD4* and *RPS20* have also been added to guideline-based testing for colorectal cancer and polyposis [44]. As to guideline-based breast cancer testing, no novel CPG additions were noted, highlighting the variable success of gene discovery studies based on ES/GS depending on cancer type [45].

The clinical approach to HCT has traditionally prioritized clinical actionability over diagnostic resolution. SR MGPT currently offers a pragmatic solution to obtain high quality sequencing data for the coding regions of clinically actionable cancer predisposition genes. Comparable analytic sensitivity needs to be established before transitioning HCT to ES/GS-based assays to ensure quality is not compromised.

Other aspects that should also be considered include the larger data footprint and increased computational requirements for storage, reprocessing, and reanalysis of ES/GS data. Importantly, this also raises the possibility of uncovering incidental findings unrelated to oncology, such as variants associated with severe rare genetic disorders.

## The utility of SOMATIC testing

Tumor-only testing is conducted on patients primarily to identify biomarkers to guide treatment selection and predict response. While it is possible to infer germline variants from somatic testing by relying on variant allele frequencies, confirmatory germline testing is recommended as allelic frequencies of true germline variants can be skewed outside the expected range (40–60%) due to several factors including tumor sample purity, somatic CNV, coverage, sequencing depth, and variant location [46]. Consequently, tumor-only sequencing is known to miss a significant number of germline variants [47].

To overcome these limitations, paired germline-somatic testing is conducted to unequivocally identify both germline and somatic variation, enabling the detection of somatic second hits, loss of heterozygosity, microsatellite instability, and tumor mutational burden [48]. In a study by Salvador MU et al., approximately 50% of patients referred for Lynch Syndrome (LS) testing, with abnormal immunohistochemistry results or microsatellite instability, were found to be double somatic mutation carriers or showing somatic *MLH1* promoter hypermethylation [8]. The authors presented examples where loss of MMR staining was explained by double somatic MMR mutations while a germline PV was identified in a different MMR gene. They further proposed that somatic tumor data may serve as supporting evidence for variant classification; however, this approach has not yet been fully integrated into established testing guidelines, including those of the ACMG/AMP or the International Society for Gastrointestinal Hereditary Tumors (InSiGHT).

In this context, somatic mutational signatures could also prove useful, as they originate with high specificity from defects in certain CPG. For example, signature SBS36 and SBS30 emerge as a result of biallelic inactivation of *MUTYH* and *NTHL1*, respectively. Likewise, *POLE/POLD1* defects result in SBS10 signatures and hypermutated tumors [49]. Identifying these signatures in patient tumors undergoing HCT could guide VUS reclassification or point to potentially undetected germline PV.

For research studies, large matched normal-tumor datasets are leveraged to uncover novel associations, or to better understand the contribution of germline PV to tumorigenesis. For instance, paired data from >34,000 patients recently suggested a potential role of *NBN* as a pan-cancer susceptibility gene [50]. Similarly, data from >17,000 patients revealed that 27% of cancers diagnosed in patients with high penetrance germline PV were not dependent on the contribution of the germline allele for tumorigenesis and occurred in tumor lineages outside the phenotypic spectrum of the corresponding CPG [51]. This aspect is particularly relevant when considering therapeutic implications, as not all cancers diagnosed in hereditary cancer patients will benefit from targeted therapies, highlighting the added clinical value of paired normal-tumor testing. In this setting, tumor retesting may reveal additional therapeutic opportunities, particularly when considering the accumulation of somatic alterations as the result of tumor evolution.

Tumor molecular testing is most commonly performed on formalin‑fixed, paraffin‑embedded tissue due to its cost‑effectiveness and suitability for long‑term preservation and storage. However, formalin fixation induces DNA fragmentation and chemical damage, which can introduce sequencing errors and artifacts [52]. Although mitigation strategies have been developed, such as S1 nuclease treatment, higher‑quality sequencing data are obtained when fresh or fresh‑frozen tissue is used as the input material. This is particularly important for LRS, as DNA integrity is a key determinant of average read length.

## Long read sequencing as an emerging approach to improve the accuracy of HCT

### LRS: technology

LRS differs from SRS primarily on the length of reads, ranging from a few kilobases to 100 s of kb, compared to ~150 bp in SRS. There are two native LRS technologies developed by Pacific Biosciences of California (PacBio; Menlo Park, CA, USA) and Oxford Nanopore Technologies (ONT; Oxford, UK). PacBio sequencing relies on circularizing DNA fragments and real time amplification with light signal detection as bases are incorporated. The polymerase performs several passes over the inserts generating multiple subreads that are then collapsed in silico into a consensus high-fidelity read. ONT sequencing relies on passing DNA libraries through nanopores to directly measure electrical signal disruptions, called squiggles, that are specific to each base. In both technologies, the kinetics of base incorporation or squiggles, i.e., the time it takes to register consecutive signals, is leveraged to robustly identify epigenetic modifications. In short, PacBio sequencing offers higher accuracy, similar to SRS, and read lengths up to ~20 kb. ONT allows longer reads reaching >10–100 s kb but suffers from higher sequencing error rates. Both technologies are continually improving through changes of reagent chemistry, nanopore/polymerase bioengineering, and/or base calling algorithm updates.

Over 50% of the human genome consists of repetitive sequence, such as mobile elements (DNA/RNA transposons) and tandem repeats, and about 7% consists of segmental duplications [53, 54]. These regions are difficult to sequence and map accurately to the right location in the reference genome with ~150 bp reads, primarily due to the high sequence homology or the repetitive/GC-rich sequence context. Additionally, repetitive regions are particularly prone to damage during DNA replication and recombination, making them susceptible to drive structural aberrations [55]. The longer reads are superior in resolving these challenging regions while also allowing robust SV identification as more sequence identity is available to delineate the breakpoints compared to SRS (Table 1). In fact, studies leveraging long-read WGS (LR-WGS) have consistently identified ~25,000 unique SV/genome compared to ~11,000 in short-read WGS data [11]. Based on these observations, SRS-based assays could easily miss SV in CPG, providing an opportunity to increase diagnostic yield.

In parallel, specialized Bioinformatic algorithms specifically tailored to LRS are also emerging and evolving. Notably, the Paraphase tool has been developed to resolve medically challenging genes such as *PMS2/PMS2CL*, facilitating the disambiguation of variation with clinical relevance without relying on orthogonal confirmation with MLPA or long-range PCR sequencing [56]. Similarly, improvements to SV/CNV calling were made, with tools like Sawfish and Sniffles2 resulting in better accuracy and speed and enabling the detection of mosaic events [57, 58].

### LRS: DNA-seq

**Fig. 1:**
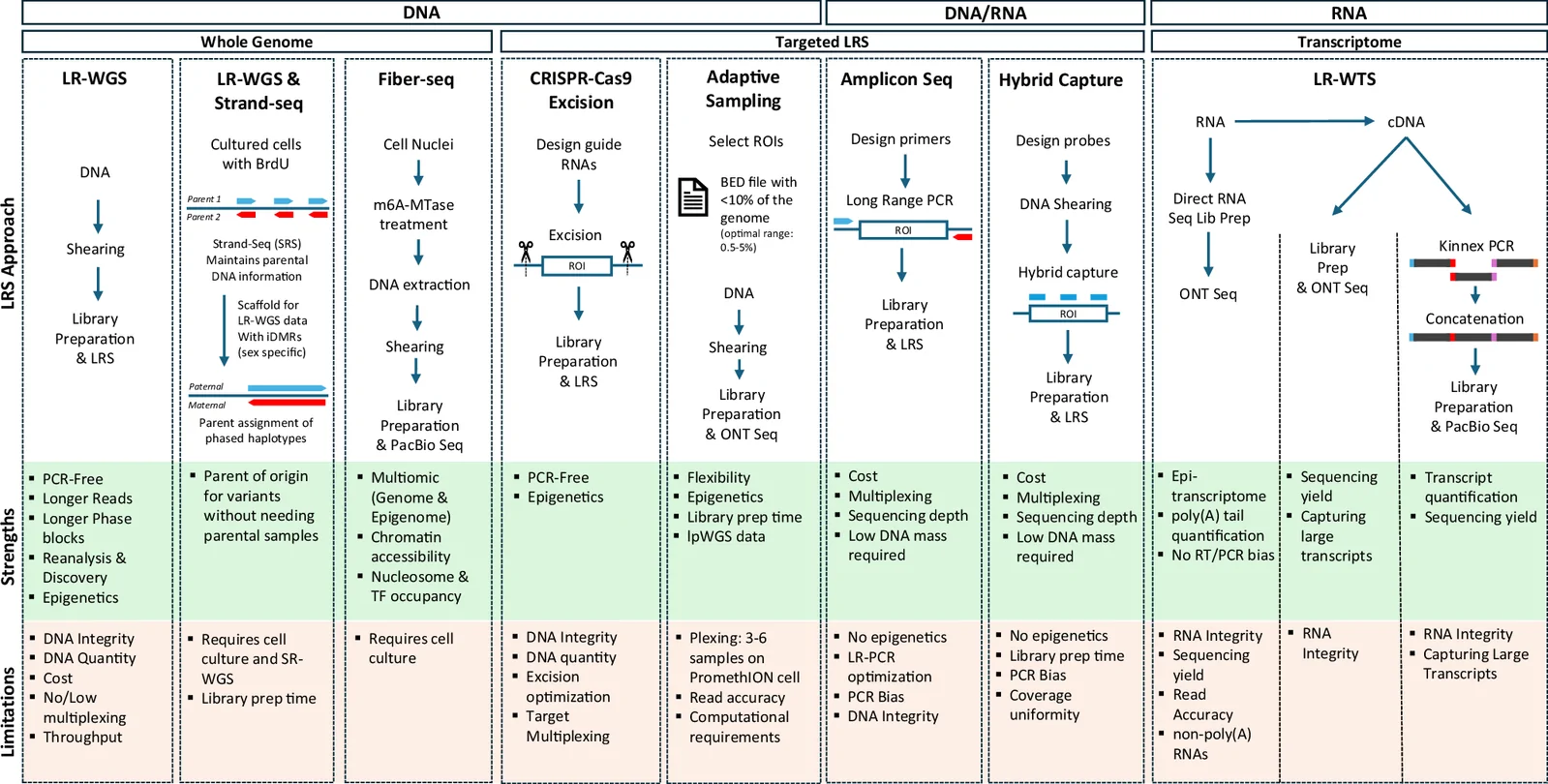
Summary of LRS approaches. Simplified workflow description, strengths and limitations. Abbreviations: BrdU: 5-bromo-2’-deoxyuridine, iDMRs: imprinted differentially methylated regions, lpWGS: low-pass WGS, m6A-MTase: *N*^6^-methyladenine methyl-transferase, ONT: Oxford Nanopore Technologies, ROI: region of interest, Seq: Sequencing.

Over the past five years, >20 research studies have leveraged LR DNA sequencing in cases with known or suspected hereditary cancer conditions (Table 2). These studies utilized different sequencing protocols including LR-WGS and targeted LRS, the latter being more cost effective and scalable thanks to the higher multiplexing. Targeted LRS approaches varied from PCR amplicon sequencing, hybridization capture, CRISPR-Cas9 excision, or ONT adaptive sampling (ONT-AS), each having different strengths and limitations that we summarized in Fig. 1. Notably, ONT-AS consists in the real-time enrichment of pre-defined genomic regions of interest, and rejection of off-targets. As DNA strands pass through the nanopore, the first few hundred bases are read, and a decision is made to continue sequencing or rejecting the strand, resulting in increased sequencing depth in the desired targets (usually 20–60x with long reads), and low-pass genome-wide coverage ( ≤ 5x with shorter reads). Interestingly, the off-target reads can be leveraged for the imputation of single nucleotide polymorphisms and showed strong concordance (99.8%) with SRS data in a recent study by Nakamura et al., enabling PRS estimation [59].

**Table 2:** A compendium of studies using LR DNA-Seq in cancer patients with known or suspected hereditary cancer conditions.

Study (PMID)	LRS Cohort	Study Type^a^	Cancer types^b^	LRS Technology	LRS Approaches^c^	Primary findings^d^
Baumann A, 2025 (40180948)	1	V	FAP	PacBio	DNA: LR-WGS	*APC* In7 LINE-1 INS (6.1 kb) that resulted in abnormal splicing.
Ohori S, 2025 (40629180)	2 (related)	D	BC	ONT	DNA: AS, Amplicon Seq; RNA: RT-PCR Seq	*BRCA1* In15 SVA INS (2.7 kb). Aberrant *BRCA1* transcript showing SVA INS ( ~ 730 bp)
Zheng Y, 2025 (40719757)	191 (9 related)	V/D	SWN	PacBio	DNA: Amplicon Seq (*NF1*)	In 186 patients (97.4%), results were concordant with ES. 51/186 (27.4%) remained negative. In 2 patients, breakpoints for 2 NF1 SV were resolved: Ex49-58 DEL (53 kb) and Ex37-40 DEL (25.8 kb). LRS refuted a previously miscalled DEL by ES. In 2 patients, LRS identified deep intronic SNVs: 1 PV, 1 VUS.
Jacobson AL, 2025 (40571398)	4 (unrelated)	D	CRC/Polyposis	ONT	DNA: AS; RNA: Direct RNA-Seq (WTS)	SV in *MSH2* (INS, 326 bp) and *APC* (INS, 6 kb). 2x VUS in *MLH3* and *MUTYH* were phased in trans with known PV. All 4 variants resulted in abnormal splicing.
Stacey AW, 2024 (39724000)	16 (unrelated)	PofO	RB	ONT	DNA: AS	PofO identification in all 16 patients enabled by phasing of PV with iDMR in *RB1* In1. Paternally inherited allele associated with higher disease severity.
Kramer M, 2024 (39005350)	10 (2 Trios, 1 Quad)	D	CRC (Trios), TGCT (Quad)	ONT	DNA: LR-WGS	Candidate SV and DMRs identified
Nakamura W, 2024 (38368425)	33 (unrelated)	V/D	Multiple	ONT	DNA: AS	In 2 FAP patients: *APC* SVA INS in In8 or In9 (2.7 kb); In 1 LS patient: Allele-specific *MLH1* epimutation; In 2 RB patients: Resolved breakpoints of *RB1* In20-3’UTR DEL (44 kb) or a balanced TRA affecting *RB1* and *LRMDA*; In 2 FAP patients: Resolved *APC* breakpoints supporting a TRA, or complex rearrangements involving INV and DEL; Identified robust SNP imputation accuracy from AS off-target reads, showing good concordance with SR-WGS (99.8%), leading to optimal PRS calculations.
Perez-Becerril C, 2024 (38302265)	1	V/D	SWN	PacBio	DNA: LR-WGS	Complex rearrangement ( ~ 15 Mb). Multiple breakpoints/breakends identified, including in *NF2*
Bjørnstad PM, 2024 (38030917)	2 (unrelated)	V	LS	ONT	DNA: LR-WGS	*MSH2* In7 INS (39 kb) in both patients
Gulsuner S, 2024 (39271294)	240 (from 120 families)	D	HBOPP	ONT	DNA: AS; RNA: RT-PCR Seq	In 8/120 families (6%): Rare deep intronic SNVs in *BRCA1, PALB2 and ATM*, that resulted in RNA pseudoexon inclusion.
Oda S, 2023 (37601671)	1	V	FAP	ONT	DNA: AS	*APC-TP63* TRA
Kumpula TA, 2023 (37578974)	2 (unrelated)	V	BC	PacBio	DNA: LR-WGS	Identified 2 events: 1) *RAD52* Ex2-9 DEL (31.5 kb) + *RAD52* In10 SVA INS (2.8 kb); 2) *RAD51C* 5’UTR-Ex7 DUP
Filser M, 2023 (37263769)	1	V	BC	ONT	DNA: AS	*BRCA1* Ex18-20 tandem DUP. Breakpoints resolved.
Dixon K, 2023 (36797466)	19 (from 18 families)	V	BC	ONT	DNA: LR-WGS	Breakpoints fully resolved for 13 SV, and partially for 1 SV, in *BRCA1, BRCA2, CHEK2 and PALB2* (510bp-108kb). Identified SV allelic heterogeneity and shared megabase long haplotypes.
Akbari V, 2022 (36777186)	5 (trios)	PofO	Multiple	ONT	DNA: LR-WGS	Parent of origin identified in all 5 trios for all autosomes with average mismatch error rate of 0.31% for SNVs and 1.89% for indels.
Watson CM, 2022 (35014072)	1	V	RB	ONT	DNA: Amplicon Seq	Resolved breakpoint of *RB1* Ex23 DEL (4 kb), unexpectedly uncovering a previously missed *RB1* Ex24 DUP (85 bp).
Paske IBAWT, 2022 (36037994)	32 (unrelated)	D	LS	PacBio	DNA: Amplicon seq	In 6/32 patients (19%): Non-coding MMR PV that resulted in abnormal splicing, including 4 deep intronic SNV/indels and 1 Alu INS.
Scharf F, 2022 (34911816)	1	V/D	(A)FAP	ONT	DNA: LR-WGS; RNA: cDNA Seq Hybrid Capture	Resolved breakpoints of multiple chromothripsis fragments. Identified 1 complex rearrangement affecting *APC* and separating promoter 1B from the rest of the coding sequencing. LR RNA-Seq identified downregulated *APC* expression.
Kwong A, 2021 (34994627)	1	V	BC	ONT	DNA: LR-WGS	*PALB2* Ex13 DUP (13.4 kb)
Sabatella M, 2021 (34231212)	2 (siblings)	V	ATRT	PacBio	DNA: LR-WGS	Precise breakpoints defined for *SMARCB1* SVA INS In2 (2.8 kb).
Yamaguchi K, 2021 (33958709)	5 (unrelated)	V/D	LS	ONT	DNA: Hybrid capture	In 4/5: Confirmed and resolved breakpoints for PV (DEL/DUP) in *MLH1/MSH2* (1.2-109 kb). In 1 case: discovered *MSH2* Ex7-16 DEL (84 kb)
Walsh T, 2021 (33060287)	19 (unrelated)	D	BC	PacBio	DNA: CRISPR/Cas9 Excision	In 1/19 patients: *BRCA1* SVA INS In13 (2.9 kb) resulting in pseudoexon inclusion
Aagaard KS, 2020 (33058509)	1	V	JPS-HHT	ONT	DNA: LR-WGS	Validated a balanced TRA (Chr1-18) affecting *SMAD4*. Precise breakpoints resolved.
Thibodeau ML, 2020 (32624572)	13 (unrelated)	V/D	Multiple	ONT	DNA: LR-WGS	In 3/13: Resolved *TSC2* INV-DUP (downgraded to LB). Event was originally miscalled by SR-WGS. In 2/13: Resolved complex rearrangements in *TSC2/NTHL1* (PV) or *NSD1* DUP (downgraded to LB). In 6/13: Confirmed PV (DEL) breakpoints in *BRCA1*, *EPCAM-MSH2*, *FANCA* or *PALB2*. In 2/13: Resolved PV (DEL) breakpoints in *ATM* or *RAD51C*.

^a^Study types: D: Discovery (Identification of new variants/alterations), PofO: parent-of-origin studies, V: Validation (confirmation or characterization of previous findings).

^b^Cancer types investigated: (A)FAP: (attenuated) familial adenomatous polyposis, ATRT: atypical teratoid rhabdoid tumor, BC: breast cancer, CRC: colorectal cancer, HBOC: hereditary breast or ovarian cancer, HBOPP: hereditary breast, ovarian, pancreatic, and/or prostate cancer, JPS-HHT: juvenile polyposis syndrome and hereditary hemorrhagic telangiectasia, LS: Lynch syndrome, RB: retinoblastoma, SWN: schwannomatosis, TGCT: testicular germ cell tumor.

^c^AS: ONT adaptive sampling, LR-WGS: long read whole genome sequencing, PCR: polymerase chain reaction, RT-PCR: Reverse transcription PCR, WTS: whole transcriptome sequencing.

^d^B/LB: benign or likely benign, DEL: deletion, DUP: duplication, (i)DMR: (imprinted) differentially methylated regions, ES: exome sequencing (short-read), Ex: exon, In: intron, INS: insertion, INV, inversion, kb: kilobases, LINE-1: Long Interspersed Nuclear Element-1, Mb: megabase, PRS: polygenic risk score, PV: pathogenic variants, SNP: single nucleotide polymorphism, SNV: single nucleotide variants, SR-WGS: short-read whole genome sequencing, SV: structural variant, SVA: SINE-VNTR-Alu, TRA: translocation, VUS: variant of unknown significance, WTS: whole transcriptome sequencing.

Validation studies aimed at using LRS primarily to confirm and fully characterize SV calls that were previously identified using other approaches such as SRS, MLPA, or Optical Genome Mapping. By using LR-WGS, Dixon et al. were able to fully resolve the breakpoints of 13/14 SV from 19 patients with sizes varying from 510 bp to 108 kb, leading to the identification of allelic heterogeneity in *BRCA1* Ex1-2 deletions (6.6-37 kb; NM_007294) and *CHEK2* Ex9-10 deletions (5.4 kb or 6.2 kb; NM_007194) [60]. A common 1.26 Mb haplotype was identified among carriers of the 5.4 kb *CHEK2* deletion, a founder in the Eastern European population, and a shared 1.08 Mb haplotype was found in three unrelated carriers of the British founder *BRCA1* Ex6 duplication (6.1 kb). Similarly, in an unexplained familial adenomatous polyposis family, Baumann et al. first identified evidence of an intronic insertion in *APC* intron 7 with SRS, and leveraged LR-WGS to fully resolve the event as a 6.1 kb LINE-1 insertion that resulted in abnormal RNA splicing [61]. In another study, Watson et al. used long-range PCR and LRS on one patient to confirm a 4 kb *RB1* Ex23 deletion (NM_000321), initially identified by MLPA, uncovering an additional 85 bp Ex24 tandem duplication that was previously missed [62].

Other studies leveraged LRS to explore missing heritability in unsolved cases with a clinical suspicion of hereditary cancer. Gulsuner et al. utilized ONT-AS in 120 unsolved kindreds affected by hereditary breast, ovarian, pancreatic, and/or prostate cancer to explore the frequency of rare deep intronic variants. The authors identified 7 PV in *BRCA1*, *PALB2* and *ATM* in 8/120 (6%) families. LR RNA-seq revealed aberrant transcripts for all 7 variants and consisted of pseudoexon inclusion resulting in premature truncation [63]. Likewise, Paske et al. utilized long-range PCR and LRS to interrogate the entire coding and non-coding regions of the MMR genes in a series of 32 patients suspected to have LS. Their analysis revealed 6/32 (19%) deep intronic *MLH1/MSH2* PV carriers, where evidence for abnormal splicing was also provided [64].

### LRS: RNA-seq

Compared to SRS, LR RNA-Seq provides multiple advantages: (1) It allows the sequencing of entire transcripts and enables the discovery of novel ones that could be associated with disease; (2) It allows the quantification of transcript levels and the transcript-aware characterization of multiple splicing events, which could have implications on variant classification; (3) in the presence of heterozygous exonic markers, it enables allele specific quantification of transcripts. Taken altogether, this provides a higher resolution when analyzing RNA data and facilitates VUS interpretation.

Schwenk et al. developed the capture and ultradeep long-read RNA seq approach (CAPLRseq), covering 123 CPG. Focusing on the diagnosis of LS, the authors validated the assay in 17 cases with PV in the MMR genes [65]. Eight patients were carriers of variants with previously available RNA data from PCR and Sanger sequencing, while nine patients were carriers of PV without previously available RNA data. CAPLRseq data was concordant with abnormal splicing and allowed the accurate identification and quantification of transcript levels. Interestingly, one of the variants was a *MLH1-DCLK3* inversion that resulted in fusion transcripts that are challenging to resolve with SRS, and another consisted in constitutional methylation (hereinafter, epimutation) affecting *MLH1*, where CAPLRseq expression levels were consistent with *MLH1* monoallelic expression. The authors also applied this method to resolve two VUS in *PMS2* and *MSH6*, successfully leading to reclassifications.

Similarly, Aucouturier et al. developed a LR-RNA target enrichment approach capturing 28 CPG compatible with both PacBio and ONT platforms. They also developed a computational framework named SOSTAR (iSofOrmS annoTAtoR) allowing the processing of raw data and the automatic annotation of transcripts in a human readable nomenclature [66]. The authors validated this approach in eight samples including four negative controls from healthy donors, two positive controls with *BRCA1* spliceogenic variants, and two relatives with abnormal *BRCA1* splice junctions suspected to have a deep intronic cryptic event. The negative controls recapitulated known *BRCA1/2* alternative splicing events and allowed a comprehensive look into transcript architecture, including the identification of novel transcripts. In positive controls, both LRS technologies correctly identified the expected aberrant *BRCA1* transcripts. For the missing heritability patients, it led to the identification of a 900 bp pseudoexon inclusion caused by a 2.7 kb SVA (SINE-VNTR-Alu) insertion leading to its classification as a PV. Noteworthy, the authors described differences in the ability of the two LRS technologies in capturing longer transcripts, with ONT being superior to PacBio. This is informative to future studies focusing on CPG with large cDNAs such as *ATM* and *BRCA2*, where selecting one technology over the other is more appropriate, and where RNA sample integrity is critical, underscoring the importance of specimen type and RNA isolation methods.

### LRS: Epigenetics

When used on native DNA molecules, LRS allows the direct detection of epigenetic modifications such as 5-methylcytosines, creating an opportunity to directly identify these marks on PCR-free LRS data. Epimutations are a well-known mechanism for cancer predisposition, albeit understudied and infrequently tested for in the clinical setting [67]. Notably, *MLH1* epimutations are known to be associated with LS. LRS can directly identify allele specific *MLH1* epimutation carriers, however, certain individuals exhibit mosaic levels of methylation ( ≤ 10%), making them harder to detect without sensitive molecular assays [59, 68]. Such events might not be detected by current PCR-free LRS approaches (LR-WGS, ONT Adaptive Sampling or CRISPR/Cas9 excision) due to the limited sequencing depth. Beyond promoter methylation in CPG, LRS could be leveraged to study epimutation signatures, such as the known episignature in Beckwith-Wiedemann syndrome, consisting in abnormal methylation patterns in the imprinted 11p15.5 region, and predisposing to embryonal tumors [69].

Additionally, methylation data from LRS can be leveraged to assign a parent-of-origin in carriers of PV without parental testing, positively impacting clinical management by simplifying cascade testing, improving variant curation and penetrance estimation. Akbari et al. described an approach based on short-read single-cell template strand sequencing (Strand-seq) to create sparse chromosome haplotypes serving as scaffolds for LR-WGS to identify imprinted differentially methylated regions, resulting in chromosome-length haplotypes and phased variant calls assigned to each parent [70] (Fig. 1).

Finally, Fiber-Seq is a recently developed LRS-based approach to study chromatin accessibility, transcription factor and nucleosome occupancy on multi kilobase chromatin fibers, enabled by the ability of LRS to detect additional epigenetic marks such as m6A (N^2^-methyladenine) [71] (Fig. 1). It provides synchronous genome and epigenome profiling, shedding light on the contribution of rare non-coding variation to human disease.

## Conclusion

Multigene panels have been the standard sequencing approach for clinical HCT for the past decade, representing a cost-effective strategy to interrogate multiple CPG simultaneously, without compromising on analytical performance [13, 19]. Despite its convenience compared to Sanger sequencing, molecular diagnostic laboratories often rely on orthogonal confirmation assays to validate observations, especially when it comes to deletions/duplications or variants in regions complicated by pseudogenes [24]. This translates into longer turnaround times, complex laboratory workflows relying on multiple complementary assays, the need to maintain personnel competency and validated equipment, and in cases of complex alterations, uncertainty in fully characterizing DNA alterations. Additionally, requirements for assay validation limit the ability to rapidly update panels as new disease genes are discovered. While these hurdles only affect a small percentage of cases that receive clinical testing, they have a high impact on clinical lab operations resulting in an increased investment of resources.

The addition of RNA-seq to DNA-only MGPT provides direct functional evidence that aids in the interpretation of DNA variants, resulting in the reduction of VUS rates. Additionally, the cascade effect of VUS reclassification was shown to impact a larger number of patients who received DNA-only testing [27]. Similarly, the addition of tumor testing enables the identification of somatic second hits, double somatic mutation carriers, and hallmarks associated with defects in CPG, such as mutational signatures or microsatellite instability [8, 49].

The dramatic decrease of ES/GS cost over time, and the development of ultra-high-throughput sequencers could trigger a paradigm shift in the future, creating an opportunity for data reanalysis and fueling research studies. However, there are important considerations prior to this potential transition. First, the lower sequencing depth of these approaches could reduce sensitivity to detect certain alterations such as CNV. Second, the cost and time to develop and validate these assays and insurance reimbursement. And lastly, the underlying short-read sequencing technology has well-known shortcomings in the detection of structural variation and variation in complex regions [11, 24].

In this context, the emergence of LRS approaches could be transformative both in the clinical and translational research settings. On one hand, it will allow clinical laboratories to consolidate and simplify orthogonal confirmation workflows, improving testing accuracy and potentially resulting in shorter reporting times for ordering clinicians. On the other hand, it opens several research fronts that could improve our understanding of the association of germline variants and increased cancer susceptibility, especially when it comes to non-coding variation and epigenetic modifications.

It is worth noting that PRS studies will benefit from the growing adoption of GS, blended ES/GS assays, and adaptive LRS, enabling the formulation of population specific PRS for individuals of non-European ancestry and ultimately advancing equity in genetic research for populations that have been historically underrepresented [9].

The clinical utility of LRS for HCT remains to be fully delineated, as the number of studies remains scarce and focused on small, highly ascertained cohorts of cancer patients (Table 2). As LRS adoption continues to increase, future studies in larger series, especially in unselected cohorts, will be pivotal in estimating its real added clinical value. Encouraging data from rare disease research has already demonstrated an increase in diagnostic rate and shorter turnaround time enabled by this technology [72]. Importantly, compared to SRS, the cost of LRS remains high and instrument throughput is considerably lower. Optimal DNA and RNA integrity and quantity requirements are also more stringent for LRS, prompting laboratories to devise alternative protocols for DNA/RNA isolation and quality control. Additionally, downstream bioinformatic workflows and tertiary analysis solutions are still maturing, and currently available catalogs of SV, tandem repeats and epigenetic modifications are emerging but remain limited to provide accurate frequency estimates of these events in the general population to inform filtering, prioritization and interpretation.

Ultimately, integrating emerging technologies into hereditary cancer testing will require careful validation and collaboration across clinical laboratories, researchers, and professional organizations. As sequencing technologies and analytical tools continue to evolve, the boundary between research and clinical testing will become increasingly seamless. Strategic investment in LRS development, data integration, and evidence generation will determine how quickly these innovations translate into tangible improvements in diagnostic precision and the future of hereditary cancer testing.
